# Beyond the Usual Suspects: Unraveling Spleen Mastocytosis in Hypersplenism Differential Diagnosis

**DOI:** 10.7759/cureus.67124

**Published:** 2024-08-18

**Authors:** Isabel Fonseca Silva, Tiago Monteiro-Brás, Inês Araújo, Beatriz Caldeira, Rui Rua Coelho, Ana Rodrigues, Iolanda Fernandes, Sara Xavier Pires, Renata Cabral

**Affiliations:** 1 Internal Medicine, Unidade Local de Saúde de Santo António, Porto, PRT; 2 Hematology, Unidade Local de Saúde de Santo António, Porto, PRT; 3 Anatomical Pathology, Unidade Local de Saúde de Santo António, Porto, PRT; 4 Anatomical Pathology, Hicislab - Dr. Rodrigues Pereira Laboratório Anatomia Patológica, Porto, PRT; 5 Dermatology, Unidade Local de Saúde de Santo António, Porto, PRT

**Keywords:** liver, speen, splenectomy, thrombocytopenia, systemic mastocytosis

## Abstract

Systemic mastocytosis (SM) poses a diagnostic challenge. This hematologic disorder involves abnormal mast cell proliferation and concurrent tissue infiltration. SM clinical presentation is not uniform, with patients displaying a wide array of symptoms related to different organ infiltration and mast cell mediators. Splenomegaly, while not typical or specific to SM, might be present from an early stage to advanced stage, especially in the presence of thrombocytopenia. Early detection is crucial for optimal patient outcomes. We present an atypical case of SM with spleen involvement in a 63-year-old male patient with a history of persistent thrombocytopenia for five years. Upon splenectomy, histological findings were compatible with infiltration with mast cells. Remarkably, the patient showed improvement and did not require additional cytoreductive therapy. This case underlines the importance of recognizing this rare presentation and highlights the potential therapeutic role of splenectomy in aggressive SM.

## Introduction

Thrombocytopenia, characterized by an abnormally low platelet count in the blood, represents a significant hematologic abnormality frequently encountered in clinical practice. Among its differential diagnosis, chronic alcohol consumption is well documented as a cause of thrombocytopenia through multiple mechanisms. Alcohol-induced bone marrow toxicity can impair platelet production by suppressing hematopoiesis and disrupting the normal maturation of blood cells [1]. Additionally, chronic alcohol use can lead to portal hypertension and hypersplenism, characterized by splenic enlargement and increased sequestration of blood cells, including platelets. This phenomenon exacerbates thrombocytopenia as the enlarged spleen traps and destroys a larger proportion of circulating platelets [2].

Systemic mastocytosis (SM), a rare malignancy characterized by abnormal proliferation and tissue infiltration of mast cells, also presents as a complex differential diagnosis for splenomegaly, particularly when leading to hypersplenism with consequent thrombocytopenia. SM results from mast cell proliferation and infiltration in different organs, sometimes leading to life-threatening complications. The disease spectrum encompasses diverse clinical presentations, ranging from more indolent forms with no organ damage to aggressive variants with multiorgan dysfunction [3].

Detection of splenic involvement often occurs through clinical examination or imaging studies, with manifestations ranging from palpable splenomegaly to cytopenias attributable to hypersplenism, highlighting the diverse clinical manifestations and systemic impact of the disease [4,5].

Given the rarity and complexity of SM, comprehensive understanding and recognition of this clinical entity are paramount for effective management and prognostic. Treatment strategies encompass a multidisciplinary approach aimed at alleviating symptoms, mitigating mast cell mediator release, and addressing organ dysfunction. Therapeutic modalities range from pharmacologic agents targeting mast cell activation to cytoreductive therapies and, in selected cases, allogeneic hematopoietic stem cell transplantation [6,7].

In this case report, we present a patient diagnosed with SM with large burden spleen involvement. This report aims to add to the existing understanding of SM and emphasize the significance of early detection of spleen involvement for better patient outcomes.

## Case presentation

**Figure 1:**
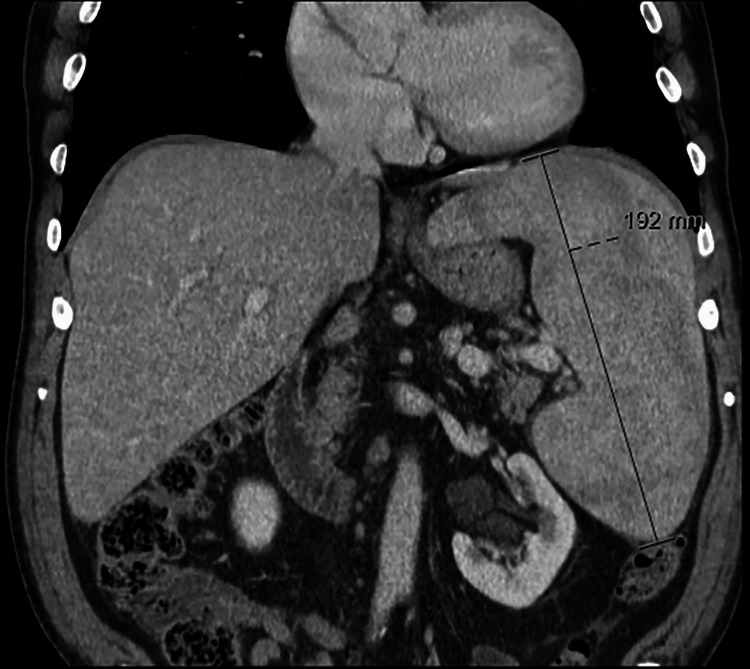
Abdominal computerized tomography scan Abdominal computerized tomography scan revealing hepatomegaly and splenomegaly with a bipolar diameter of 192 mm, exhibiting normal morphology (coronal view).

A 63-year-old male was referred to a hepatology appointment due to sustained mild thrombocytopenia over the last five years. Platelet levels varied between 100 000 and 120 000/µL (normal range: 150 000-450 000/µL). The patient displayed no history of bleeding. For medical background, the patient had hyperuricemia, arterial hypertension, benign prostatic hyperplasia, and a history of alcohol consumption since his youth with a daily intake of approximately 100 grams. On physical examination, the patient appeared well-nourished with no signs of jaundice or hepatic encephalopathy. Abdominal examination revealed a palpable splenomegaly of 4 cm below the costal margin with no other palpable organomegalies or masses. There were no stigmas of chronic liver disease. The remainder of the physical examination, including cardiovascular and respiratory systems, were unremarkable. Serological tests for viral hepatitis and autoimmune liver disease were negative. Abdominal computerized tomography (CT) demonstrated hepatomegaly with no signs of advanced liver disease, and splenomegaly with a bipolar diameter of 192 mm with a normal morphology (Figure 1). Doppler ultrasound of the hepatic vasculature showed normal flow patterns without evidence of portal hypertension. 

Given the history of alcohol consumption, alcohol-induced thrombocytopenia was suspected since liver enzymes were also increased, and a supervised abstinence program was started. However, after one year of abstinence, and the normalization of liver enzymes, a worsening thrombocytopenia, with a minimum platelet count of 22 000/µL, was still persistent, while no other cytopenias were present. During this time, a bone marrow aspirate was also performed, revealing normal cellularity and the presence of some non-atypical mast cells. Flow cytometry of the bone marrow indicated immunophenotypic alterations in the granulocytic lineage consistent with dysplasia. These dysplastic changes were interpreted in the context of alcohol consumption. It is important to note that this study was not specifically directed toward investigating mastocytosis.

Due to worsening splenomegaly, a non-invasive portal hypertension study was conducted; upper gastrointestinal endoscopy showed no evidence of esophageal varices, and hepatic elastography indicated a fibrosis stage of F0-F1, indicating mild fibrosis but no evidence of cirrhosis. Invasive hepatic pressure measurements revealed a hepatic venous pressure gradient of 8 mmHg, suggesting clinically insignificant portal hypertension and therefore not justifying the observed splenomegaly. A liver biopsy confirmed hepatic siderosis, consistent with alcoholic liver disease with no signs of cirrhosis. 

**Figure 2:**
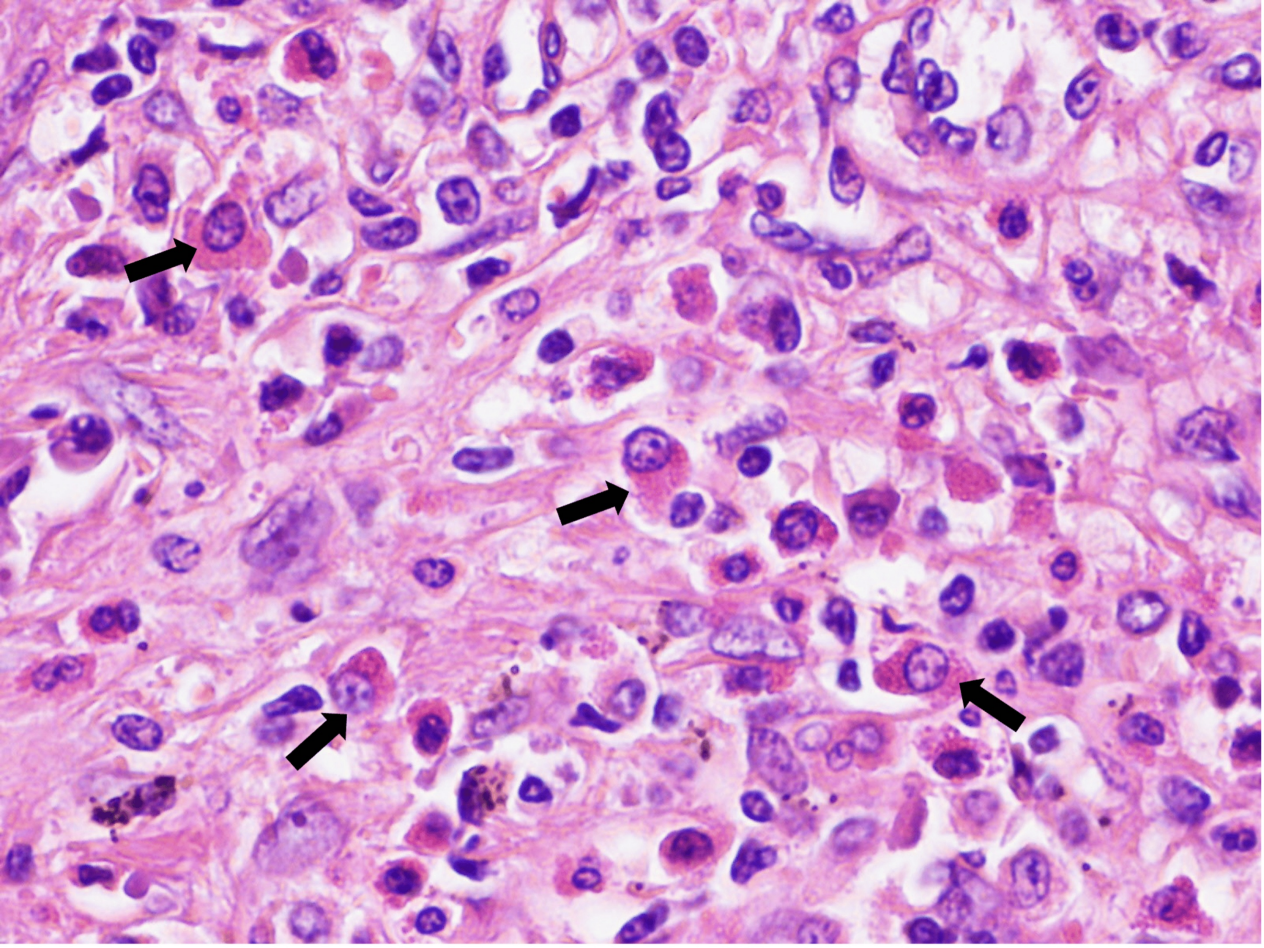
Histological examination of the spleen Tissue of spleen depicting several eosinophilic mast cells (hematoxylin and eosin, 400x)(black arrow)

**Figure 3:**
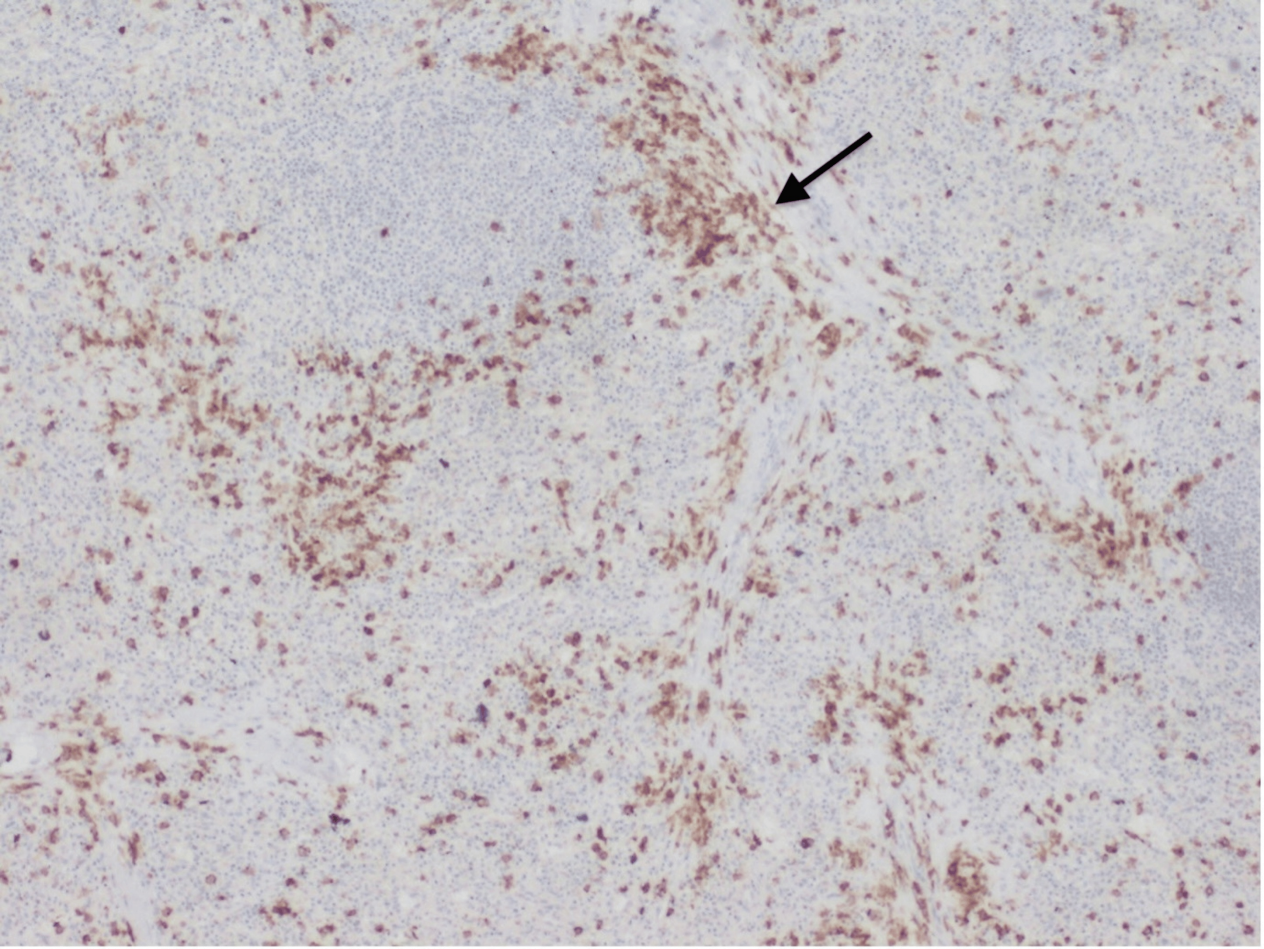
Immunohistochemical study of the spleen Spleen highlighting irregularly dispersed mast cells infiltrate with strong CD117 expression (CD117, 40x)(black arrow)

**Figure 4:**
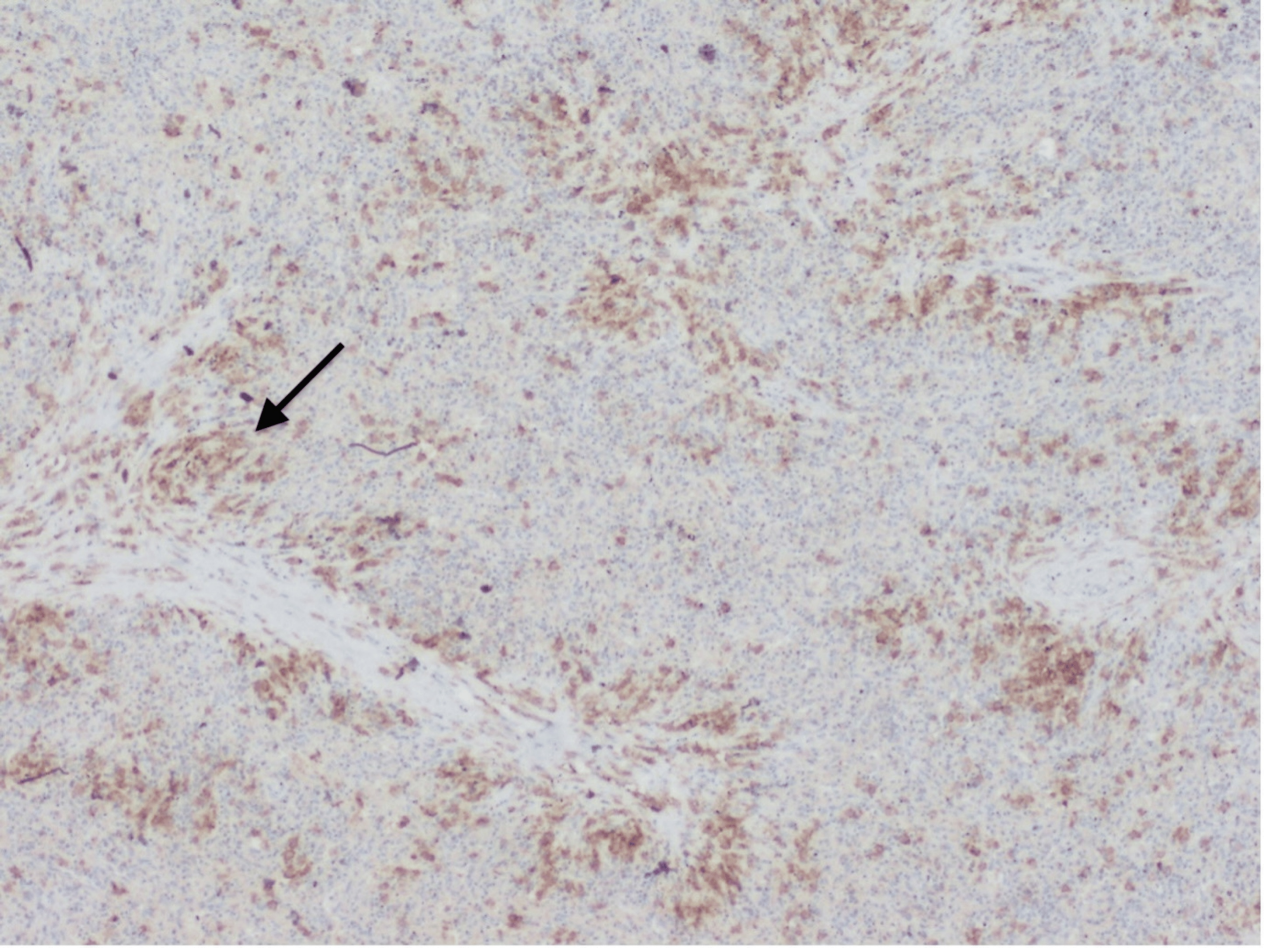
Immunohistochemical study of the spleen Spleen highlighting irregularly dispersed mast cells infiltrate with aberrant expression of CD25 (CD25, 40x)(black arrow)

During this time, the patient experienced progressive weight loss and localized left hypochondrium pain that did not respond to analgesia, alongside progressive splenomegaly and sustained thrombocytopenia despite the absence of hemorrhagic events. Following a multidisciplinary discussion involving hepatology, hematology, and general surgery, splenectomy was done with no complications, and the patient was monitored for potential complications of surgery, including bleeding and infection. The histological examination of the spleen showed the presence of aggregates of small to intermediate cells (Figure 2). The immunohistochemical study revealed positivity for CD117 and CD25 suggesting splenic involvement by mastocytosis (Figures 3, 4).

**Figure 5:**
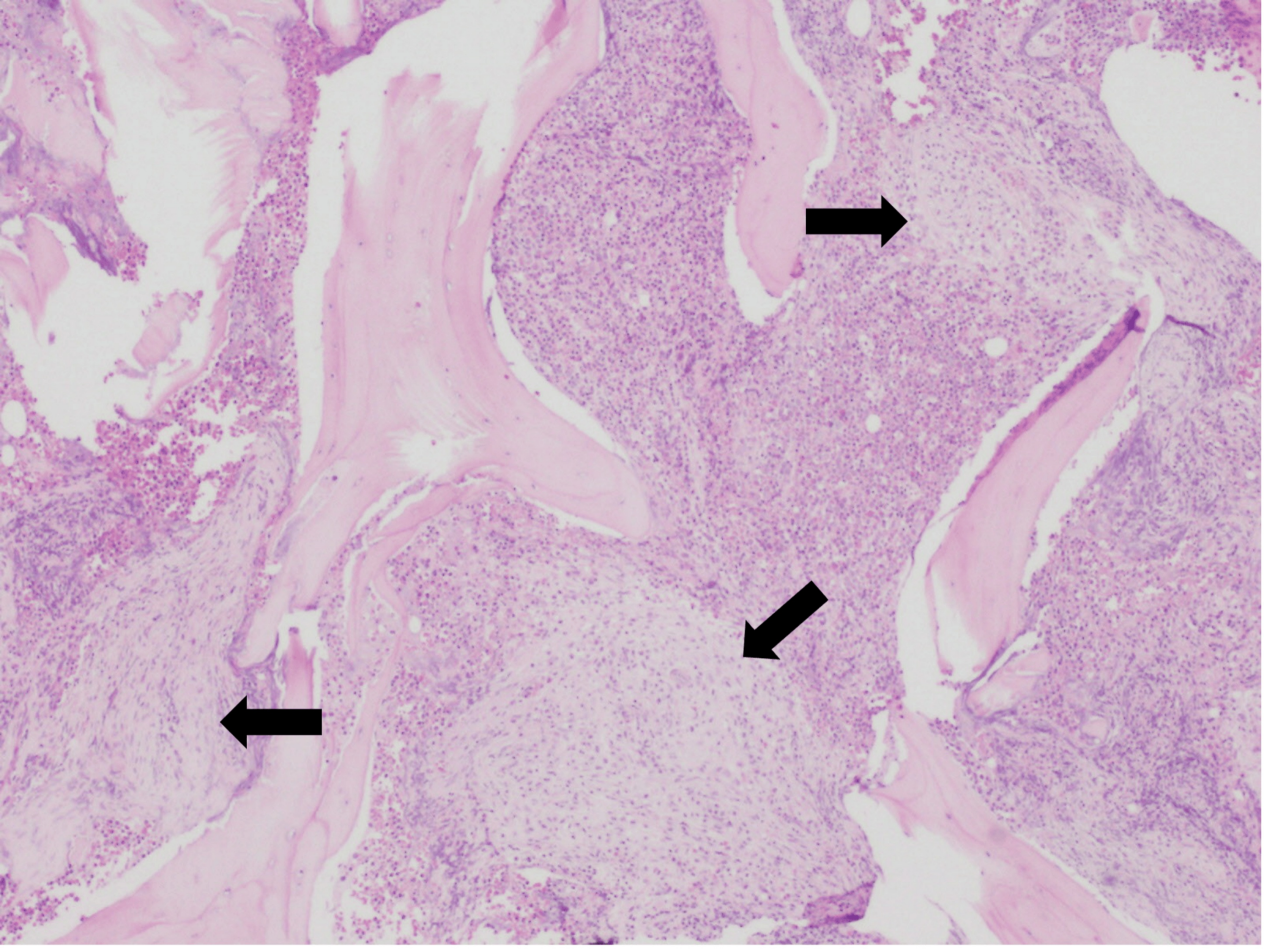
Histological examination of the bone marrow Bone marrow biopsy (decalcified) showing multifocal aggregates of pale staining mast cells (hematoxylin and eosin, 40x)(black arrow)

**Figure 6:**
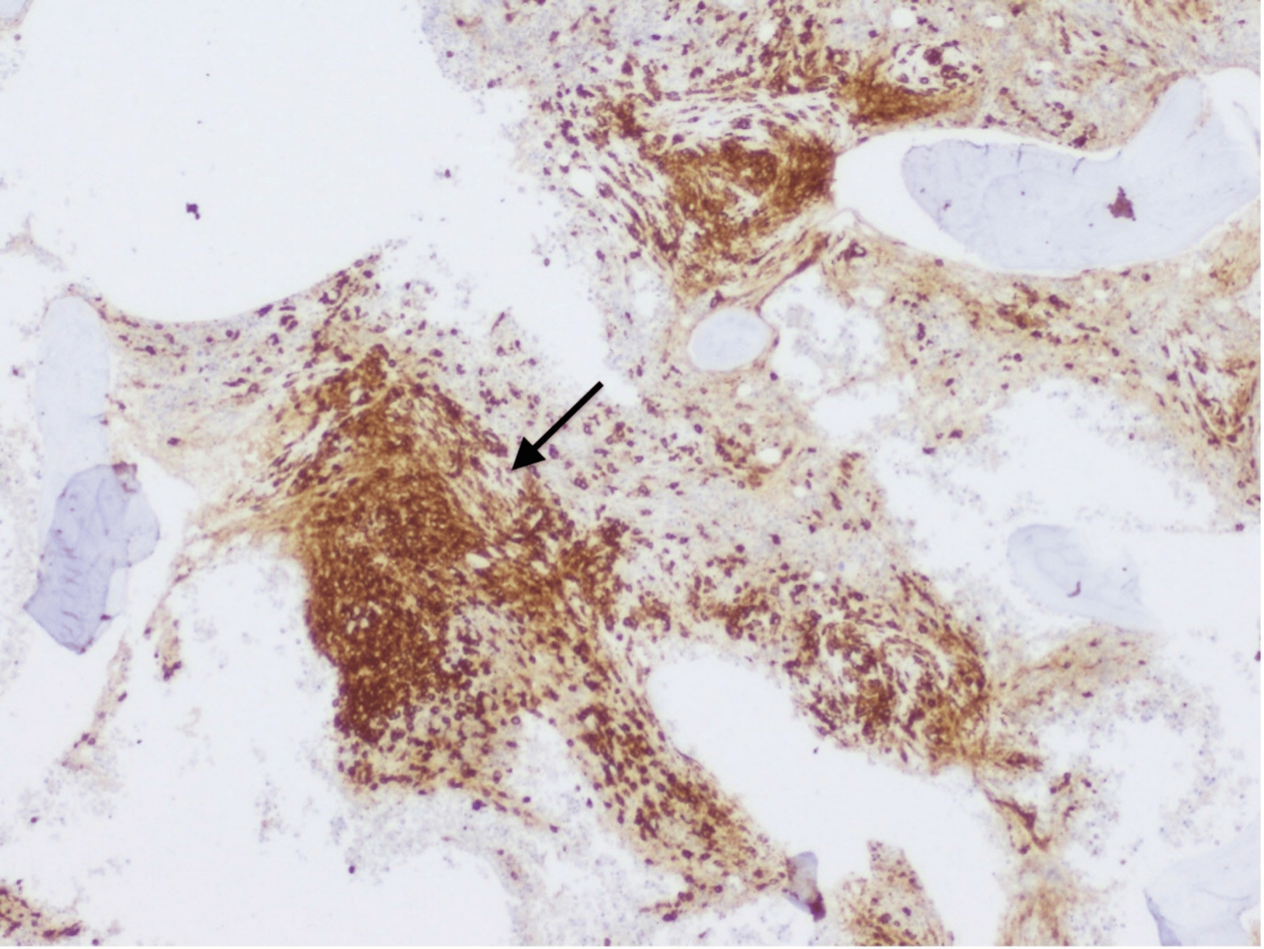
Immunohistochemical study of the bone marrow Bone marrow biopsy shows dense aggregates of mast cells, with strong CD117 expression (CD117, 40x)(black arrow)

**Figure 7:**
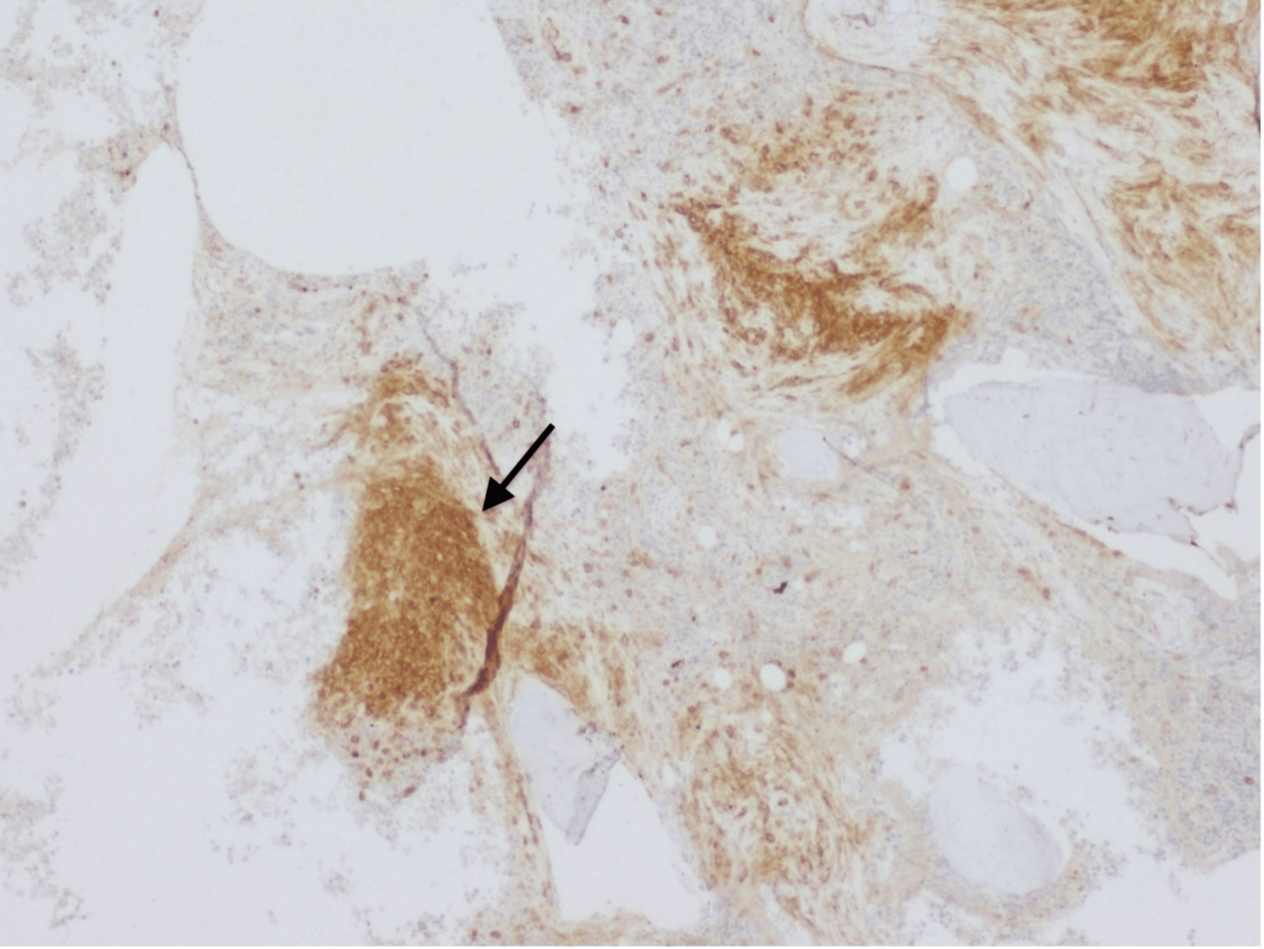
Immunohistochemical study of the bone marrow Bone marrow biopsy shows aberrant expression of CD25 in aggregates of neoplastic mast cells (CD25, 40x)(black arrow)

At the same time, a bone marrow biopsy was performed with histology showing frequent mast cells, with aggregates of more than 15 mast cells identified, representing 40% of cellularity, with approximately 30% of spindle-shaped mast cells (Figure 5). The bone marrow immunohistochemical study showed positive markers for tryptase, CD117, CD25, and CD30, with CD2 positive in a few cells (Figures 6, 7). 

Considering the findings described above, a comprehensive review of associated symptoms was conducted. The patient experienced gastrointestinal symptoms such as flatulence and occasional diarrhea after consuming certain foods. There were no documented allergic reactions or episodes of anaphylaxis. Dermatological assessment revealed an absence of lesions or flushing. Neurologically and psychiatrically, there was no history of cognitive impairment or mood disturbances. Blood samples were collected to measure serum tryptase levels, which were found to be elevated at two separate measurements taken approximately five months apart, with levels recorded at 125 μg/L and 194 μg/L (normal value below 11.4µg/L), respectively, while serum IgE and albumin levels remained normal. Moreover, the presence of the KIT D816V mutation was confirmed in both peripheral blood and bone marrow samples. 

A diagnosis of SM was made, and the patient started therapy with antihistamines, with a complete resolution of thrombocytopenia after splenectomy and complete resolution of the previous symptoms, with no other symptoms, thereafter, regaining his usual weight. A bone densitometry scan was performed, revealing bone density values within normal parameters. As the patient had no additional C criteria (of organ failure) after splenectomy, which indicated the need for additional cytoreductive treatment, he was treated only with antihistamines maintaining a favorable evolution. Close monitoring was maintained for signs of disease progression that could indicate the need to initiate systemic treatment.

## Discussion

Mast cells are a type of immune cell derived from bone marrow progenitors, primarily found in connective tissues, particularly those near blood vessels and mucosal surfaces [3]. They play a crucial role in allergic and inflammatory responses, as well as in the regulation of immune function and tissue homeostasis [8]. SM is characterized by the abnormal proliferation and accumulation of mast cells in various tissues and organs. Genetic mutations, particularly in the KIT proto-oncogene, drive the clonal expansion and activation of mast cells. In fact, KIT D816V mutation is found in 95% of SM patients and results in constitutive activation of the KIT receptor tyrosine kinase, driving mast cell growth, survival, and activation, contributing to the pathogenesis of SM [9].

The abnormal proliferation of mast cells in SM results in the accumulation of mast cell aggregates, or infiltrates, in various organs, namely the bone marrow, skin, gastrointestinal tract, liver, spleen, and lymph nodes. These mast cell infiltrates can disrupt normal tissue architecture and function, leading to a wide range of clinical manifestations and organ dysfunction [10].

The release of inflammatory mediators from activated mast cells is a central feature of SM pathology. Mast cells contain granules filled with biologically active substances, including histamine, tryptase, leukotrienes, prostaglandins, and cytokines, which are released upon activation. These mediators exert pleiotropic effects on surrounding tissues and cells, resulting in vasodilation, smooth muscle contraction, increased vascular permeability, tissue edema, and recruitment of inflammatory cells [10].

Splenic involvement may precipitate a cascade of clinical sequelae, including splenomegaly, hypersplenism, and potential complications such as portal hypertension or splenic rupture, posing significant diagnostic challenges to clinicians [11]. Thrombocytopenia in splenic involvement of mastocytosis results from several mechanisms, including hypersplenism due to splenomegaly, direct suppression of hematopoiesis by infiltrating mast cells or increased sequestration and destruction of platelets by activated mast cells. The degree of thrombocytopenia may vary depending on the extent of splenic involvement and mast cell burden within the spleen [5].

The clinical manifestations of SM are diverse and can vary widely depending on the location and extent of mast cell infiltration, as well as the degree of mast cell activation. Common symptoms include skin lesions (mainly maculopapular cutaneous mastocytosis) and mainly mediator-related symptoms like pruritis, flushing, diarrhea, nausea, vomiting, abdominal pain, brain fog, hypotension, headache, fatigue, and bone pain [12]. Severe systemic symptoms, such as anaphylaxis, may occur in some cases, particularly in response to triggers such as physical exertion, emotional stress, certain foods, medications, insect stings, or other environmental factors [13].

The diagnosis of SM relies on a combination of clinical, histological, and molecular criteria established by the World Health Organization (WHO) and the European Competence Network on Mastocytosis. The diagnosis of SM may be made if one major and one minor criterion, or if three minor criteria are met [14].

Patients afflicted with SM commonly experience symptoms triggered by mediator release and/or exhibit signs arising from mast cell infiltration. The WHO has outlined distinctive clinical findings, referred to as B- and C-findings, to stratify SM classification. B-findings denote organ involvement sans organ failure, whereas C-findings signify organ involvement alongside organ dysfunction. The presence of a C-finding alone is adequate for diagnosing advanced SM [5,15].

In this case, the patient presented with criteria for SM, including one major criterion with an infiltrate of tryptase and CD117 positive mast cells (15 mast cells in aggregates) detected in bone marrow and four minor criteria: 30% of the mast cells exhibited spindle-shaped morphology in bone marrow biopsy; presence of mast cells in spleen expressing CD25 and CD2, persistent elevated serum tryptase and the presence of the KIT D816V mutation. As such, a diagnosis of SM was confirmed. C-findings were evident, including thrombocytopenia and splenomegaly with hypersplenism, indicating a SM with aggressive features. 

In SM, most patients present with cutaneous involvement, often leading to the diagnosis through evidence of mastocytosis in the skin. In adults, this usually indicates systemic disease. This particular case is challenging due to the absence of typical cutaneous lesions and symptoms of mast cell activation, such as anaphylaxis to wasp venom, pruritus, or flushing, which would typically suggest a mast cell disorder. Usually, SM is not often considered in differential diagnosis of splenomegaly, unless accompanied by other signs or symptoms indicative of mast cell pathology. Here, we present a patient with SM-associated organ dysfunction, driven by splenic mast cell infiltration [5,16].

Treatment of aggressive SM typically involves a multidisciplinary approach. Patients are advised to start histamine inhibitors to alleviate symptoms, as well as carry an EpiPen for the risk of an anaphylactic shock. Disease control may include chemotherapy to control mast cell proliferation and, tyrosine kinase inhibitors targeting KIT mutation, such as midostaurin or avapritinib, as the mutation is the main driver of the disease [17]. Hematopoietic stem cell transplantation can be seen in some specific cases. All patients should have regular medical monitoring to assess disease progression and adjust treatment as needed [18,19].

Splenectomy is typically not considered a treatment for SM. The literature regarding the role of splenectomy in the treatment of aggressive SM is limited, with only a few reported cases [20]. We report a patient with aggressive SM whose condition was effectively controlled following a splenectomy. It is important to note that this therapeutic decision was made without prior knowledge of the diagnosis of SM. If the diagnosis had been known at the time, cytoreductive therapy would likely have been considered the preferred treatment. This highlights the importance of accurate and timely diagnosis in determining the most appropriate therapeutic strategy.

Further research is needed to better understand the role of splenectomy in the management of this condition and to identify optimal treatment strategies for affected patients. Despite the patient’s history of chronic alcohol consumption, a common cause of thrombocytopenia, the underlying main etiology for the patient's low platelet count was found to be SM. 

## Conclusions

The diagnosis of SM in this case was incidental and unexpected, emphasizing the necessity of a comprehensive differential diagnosis when evaluating patients with splenomegaly and secondary thrombocytopenia. It highlights the importance of considering rare conditions like SM in cases of unexplained splenomegaly, even in the absence of classical symptoms.

Early detection and accurate diagnosis of such rare disorders are crucial for appropriate management and improved patient outcomes. Bone marrow examination, including biopsy, is therefore fundamental in the evaluation of splenomegaly of unclear etiology, as it can uncover underlying conditions that might not be otherwise apparent.
